# Diseases of the Respiratory Passages

**Published:** 1896-02

**Authors:** 


### BOOK NOTICES.

DISEASES OF THE RESPIRATORY PASSAGES. By Charles Porter Hart, M. D., with 117 illustrations. Second edition; re-written and enlarged. 450 pages. New York: A. L. Chatterton & Co., 78 Maiden Lane, 1895. Price, cloth, postpaid, $3.00.

This volume represents the experience of the author as a throat specialist along homœopathic lines of treatment, combined with the experience of others in the homœopathic school. Most reliance seems to be placed upon operative procedures, though copious homœopathic indications are given, copied from such authorities as Jahr, Lippe, Guernsey, and many others.

Looking over the pages of the book we notice many interesting things. Thus, in cases of nose-bleed, the author gives copious indications for the homœopathic remedies, mainly upon the authority of Dr. H. N. Guernsey. He also speaks of the use of Lupulin, first trituration, as an almost infallible remedy. This medicine was suggested by the experience of Dr. Parkhurst, who failed to stop a case of nasal hemorrhage; whereupon the patient took the advice of a neighbor, and snuffed powdered hops with immediate success. The author disapproves plugging the posterior nares as formerly practiced, and gives a method of doing it from the front. This method may be described as follows:

"Roll between the thumb and fingers a lock of cotton into a cylinder or little roll an inch or an inch and a half in length; tie a strong thread to the middle of the roll, bring the two ends of the roll together, and then opening the nasal orifice by pressing down with the end of the finger its lower margin, pass the middle or folded part of the roll (where the string is tied) into the nostril; next with the blunt end of a lead-pencil or stick, press in the cotton-roll slowly along the floor of the nostril an inch or more and rest. If the blood passes down into the throat you may be sure the bleeding spot is behind the roll, so push in your roll further and the blood will cease to pass behind. Then, holding on to the string, pass some loose cotton into the nostril and push it in, along with the pencil, down to the plug. The cotton will swell with moisture, compress the bleeding surface, and arrest the hemorrhage."

He also disapproves of the nasal douche. In suppuration of the mastoid cells he prefers to puncture the bone and let out the accumulated pus, and illustrates a neat little drill for accomplishing the perforation. This affection he regards as purely surgical. The writer of this review is reminded of a case of this kind occurring in his own practice for which no surgical procedures were deemed sufficient. The indicated remedy, Zincum-metallicum, was given with quick discharge of the pus through the ear canal, and complete and prompt recovery. The author recommends some alternations of medicines, especially in cases of parotitis (see page 98). This is hardly scientific.

A number of indications for quinsy are given, to which we suggest that several other indications may be added, especially Lycopodium. The inhalation of steam of hot water is also recommended. To this there can be no objection. In the reviewer's own practice he has had large experience with acute quinsy, and has had much success, except where there was found much congenital hypertrophy. The author also quotes cases by Dr. Burt where suppuration of the tonsils was arrested in three or four hours' applications of Mercurius-corrosivus, first decimal trituration, directly to the tonsil by means of a camel's hair-brush.

Follicular tonsillitis is not treated of as a separate disease in this work, but included under the term follicular sore throat, follicular pharygitis, or clergyman's sore throat. In the course of his discussion of this subject, the author manages to give the brethren of the old school a vicious little jab of his pen at page 139, where, in speaking of the use of the atomizer to apply " mineral waters rich in Natrum-carb.," he says: "We, of the progressive homœopathic school, ought to feel pleased that atoms begin to be considered of importance by all classes of physicians. When in the atomization of liquids one drop of a very weak medicinal solution is divided into thousands of invisible and imponderable atoms in order to act a remedial part, then it needs only one small step to become a convert to the beneficial action of dilutions."

Some space is devoted to intubation of the larynx, with directions for employing it.

There are but few remedies for croup given. This is to be regretted, since our most brilliant successes have been made upon croup.

We can ourselves look back upon many a hard-fought case of croup, where success came from the selection of the similar remedy.

In treating of diphtheria, the author does not appear to have included among his remedies Hydrastis and Phytolacca, which are of high importance. The writer of this notice has cured a perfectly desperate case of diphtheria with Phytolacca. The author expresses sound views upon diphtheria when he says: "There cannot be a reasonable doubt that diphtheria is just as much a constitutional or blood disease as rheumatism, scrofula, variola, or syphilis." He then gives his reasons for his view, and rather discredits the bacteria theory.

